# Do Children With Comorbid Reading and Mathematics Difficulties Experience More Internalizing Problems?

**DOI:** 10.1177/00222194251335313

**Published:** 2025-04-28

**Authors:** Ana Paula Alves Vieira, George K. Georgiou, Yuliya Kotelnikova

**Affiliations:** 1 University of Alberta, Edmonton, Canada

**Keywords:** learning difficulties, reading difficulties, mathematics difficulties, internalizing problems, anxiety, depression

## Abstract

We examined whether children with comorbid reading (RD) and mathematics (MD) difficulties experience more internalizing problems (anxiety, depression, somatic complaints, and social withdrawal) than children without comorbidity. In addition, we examined whether any significant group differences are due to differences between groups in attention. Thirty-three Canadian children with RD (51.5% female; *M*_age_ = 10.80 years), 35 with MD (60.0% female; *M*_age_ = 10.79 years), 37 with comorbid RDMD (45.9% female; *M*_age_ = 10.79 years), and 42 chronological-age (CA) controls (64.3% female; *M*_age_ = 10.82 years) were assessed on reading, mathematics, general cognitive ability, and attention tasks. Their teachers also rated their anxiety, depression, somatic complaints, and social withdrawal. Results of analyses of variance showed that children with comorbid RDMD exhibited significantly higher levels of anxiety and depression compared only to the CA controls. However, after controlling for attention, these group differences were no longer significant. These findings suggest that children with comorbid RDMD may be at greater risk for anxiety and depression, although attention difficulties likely contribute to these differences.

About 5% to 15% of the student population experiences persistent academic struggles in reading, writing, or mathematics (American Psychiatric Association [APA], 2022; Grigorenko et al., 2020). In addition to cognitive factors such as phonological awareness and approximate number system that have been found to underlie reading and mathematics difficulties, respectively (e.g., De Smedt et al., 2013; Hulme & Snowling, 2014), individuals’ attitudes and feelings toward reading and mathematics can also influence their performance.

Theoretical models such as the Componential Model of Reading (Aaron et al., 2008) or the Debilitating Anxiety Model (Hembree, 1990) have suggested that affective factors can exert an independent contribution to academic achievement and consequently underlie learning difficulties (LD) (see Note 1). In line with the proposition of these theoretical frameworks, a recent meta-analysis (Vieira et al., 2024) reported an effect size of *g* = -.54, with LD children having more internalizing problems than their chronological-age (CA) controls. However, previous studies examining the relation between LD and internalizing problems have at least three important limitations.

First, most empirical studies have been conducted with individuals having a general LD (e.g., Amitay & Gumpel, 2015; Sideridis, 2007) or with individuals having reading difficulties alone (RD; e.g., Knivsberg & Andreassen, 2008; Willcutt & Pennington, 2000b). In addition, these studies have focused on anxiety and depression (see Vieira et al., 2024, for a systematic review). Thus, it remains unclear if reading (RD) and mathematics difficulties (MD) are also associated with somatic complaints or social withdrawal. Second, only a handful of studies have examined if comorbidity in RD and MD increases the risk of experiencing internalizing problems (Aro et al., 2022; Martínez & Semrud-Clikeman, 2004; White et al., 1992; Willcutt et al., 2013) and they have their own limitations (see below). Finally, even though previous research has shown that inattention underlines both LD and internalizing problems (e.g., Aro et al., 2024; Carroll et al., 2005), only a few studies have controlled for it. Thus, the purpose of this study was to examine if children with RDMD experience more internalizing problems (anxiety, depression, somatic complaints, and social withdrawal) than children with either RD or MD, or children with no difficulties. In addition, we examined whether any significant group differences are due to differences between groups in attention.

## LD Type and Internalizing Problems Type

Data-driven methods suggest that mental health symptoms can be captured by two broad dimensions: internalizing and externalizing (Achenbach et al., 2016; Georgiou & Parrila, 2023). In particular, at the core of the internalizing spectrum is elevated negative emotionality (i.e., fear, anger, sadness), and symptoms may manifest as anxiety, depression, somatic complaints, or social withdrawal (Achenbach et al., 2016). Internalizing problems have been historically understudied because these issues are less visible when compared to externalizing problems, which are inherently disruptive (Achenbach et al., 2016). The relationship between LD and externalizing problems is well-established, with research showing that externalizing behaviors are related to deficits in reading and mathematics (e.g., Kremer et al., 2016; Nelson et al., 2004). In contrast, research on the association between LD and internalizing problems has been gaining traction more recently (e.g., Georgiou et al., 2024; Vieira et al., 2024).

Anxiety represents a learned negative emotional response to anticipation of a future threat that is maintained through avoidance (Mineka & Zinbarg, 2006). In this study, we focused on generalized anxiety, characterized by chronic, uncontrollable worries across many aspects of life, occurring for an extended period and interfering with daily functioning (APA, 2022; Gale & Millichamp, 2016). Depression often co-occurs with anxiety due to shared heightened negative emotionality. At the same time, low mood (or low positive emotionality) represents the unique emotional core of depression (Clark, 2005). Furthermore, both somatic symptoms (e.g., fatigue, aches, pains) and withdrawal from social interactions could be viewed as consequences of either anxiety or depression. Notably, the variance in somatic symptoms has been captured by a related, yet distinct, dimension in both child (Achenbach et al., 2016) and adult (Kotov et al., 2017) models of psychopathology across the lifespan.

In their recent systematic review examining individuals with LD and internalizing problems, Vieira et al. (2024) reported that 61 of the 96 studies included in the systematic review had participants with RD, 31 studies did not specify the type of LD, and only 12 studies included participants with MD. In addition, most studies investigated anxiety (*n* = 62), followed by depression (*n* = 27), somatic complaints (*n* = 16), and social withdrawal (*n* = 9). Research suggests a bidirectional relation between RD and anxiety (e.g., McArthur, 2022; Parhiala et al., 2015), as well as between MD and anxiety (Carey et al., 2016). However, only a few studies have examined the relation between MD and internalizing problems other than mathematics anxiety (e.g., Wakeman et al., 2023; Willcutt et al., 2013). Regarding the association between depression and LD, empirical studies comparing groups with and without LD have shown inconsistent findings, possibly due to heterogeneity across studies (Morte-Soriano & Soriano-Ferrer, 2024), but there is evidence that this relation might also be bidirectional (Zhang et al., 2019). Research suggests that MD can hinder daily activities requiring mathematical skills (e.g., budgeting or shopping), potentially contributing to school dropout and reduced employment opportunities, which may lead to depression in adulthood (Graefen et al., 2015). Similarly, studies indicate that childhood RD is prospectively linked to lower educational attainment and income (McLaughlin et al., 2014), as well as an increased risk of depression in adulthood (Eloranta et al., 2019). Overall, it is possible that youth with LD experience difficulties with self-concept and self-esteem formation due to their learning challenges and possibly as a result of peer victimization (e.g., Baumeister et al., 2008), prospectively leading to higher levels of internalizing symptoms (e.g., Donolato et al., 2022).

Preliminary evidence also suggests that children and adolescents with RD experience higher levels of somatic complaints (e.g., Arnold et al., 2005; Willcutt & Pennington, 2000b). Willcutt and Pennington (2000b), for example, showed that children and adolescents with RD exhibited significantly higher levels of internalizing disorders, including somatic complaints, than their co-twins and a community sample of individuals without RD. According to Willcutt and Pennington (2000b), children with RD might develop headaches and stomach aches due to school stress, which might be related to increased anxiety. To our knowledge, only two studies have investigated the relation between MD and somatic complaints (Martínez & Semrud-Clikeman, 2004; Wakeman et al., 2023). Wakeman et al. (2023) examined the association between MD and diverse internalizing problems, including somatic complaints. Although they found that adolescents with MD exhibited higher levels of anxiety and depression, this was not the case for somatic complaints. Martínez and Semrud-Clikeman (2004) found similar results for all types of LD groups (MD, RD, RDMD) on a somatic complaints measure when compared with CA controls and children with a single difficulty (MD or RD).

Although Martínez and Semrud-Clikeman (2004) did not find a significant effect of group on somatic complaints and did not test social withdrawal, they did find that the RDMD showed higher levels of social-emotional difficulties, including somatic complaints, anxiety, atypicality, locus of control, and social stress scales. Social withdrawal has not been examined in comorbidity studies yet, but there is some evidence showing that it is associated with LD. Vieira et al. (2024) found that individuals with LD presented higher scores in social withdrawal when compared with anxiety or somatic complaints. Interestingly, the relation between social withdrawal and LD was found to be different from the other internalizing problems, suggesting that it predicts academic achievement (Hall et al., 2016; Lim & Kim, 2011). In Hall et al.’s (2016) study, withdrawal in kindergarten and first grade predicted reading achievement in second grade. Such withdrawal behaviors can divert children’s attention from instruction and classroom activities, hindering their learning and contributing to delayed reading acquisition and lower academic performance (Lim & Kim, 2011).

## Comorbidity RDMD and Internalizing Problems

According to Moll et al. (2014), between 11% and 70% of children with one type of difficulty (e.g., RD or MD) also have a comorbid deficit in the other learning domain. There are two main explanatory approaches for this comorbidity: the three independent disorders model and the multiple-deficit model (Viesel-Nordmeyer et al., 2023). The three independent disorders model proposes that RD, MD, and RDMD are separate disorders, each with a unique cognitive profile (e.g., Skeide et al., 2018). In contrast, the multiple-deficit model (e.g., McGrath et al., 2020; Pennington, 2006) postulates that LD arise from complex interactions between specific core deficits unique to each disorder and shared risk factors, such as memory, attention, and processing speed (e.g., Moll et al., 2016; Raddatz et al., 2017; Slot et al., 2016; Willcutt et al., 2013). In other words, domain-specific deficits underlying RD (e.g., phonological processing) and MD (e.g., number sense) are combined, resulting in an additive profile observed in children with RDMD (e.g., Peters et al., 2020; Wilson et al., 2015).

The high rate of RDMD comorbidity highlights the need for a deeper understanding of the relationship between these two learning difficulties and their impact on children’s mental health. To our knowledge, only four studies (Aro et al., 2022; Martínez & Semrud-Clikeman, 2004; White et al., 1992; Willcutt et al., 2013) have compared RD, MD and RDMD groups in internalizing problems, and their findings are mixed. Aro et al. (2022) analyzed percentages of children diagnosed with learning disabilities with behavioral-emotional symptoms and found that more than 37% of children exhibited affective (e.g., depression) and anxiety problems, irrespective of the learning disability type. This study used parents’ and teachers’ reports. In line with this finding, Martínez and Semrud-Clikeman (2004) compared students from Grades 6, 7, and 8 with multiple and single LD on psychosocial functioning self-report measures and found that the RD, MD, and RDMD groups did not differ from each other on any measure, including anxiety, depression and somatic complaints, although the RDMD group reported the highest scores. In contrast, White et al. (1992) assessed adolescents with specific arithmetic disabilities, specific reading disabilities and both arithmetic and reading disabilities, and found group differences in anxiety measures with the comorbidity group presenting higher anxiety levels when compared to the single disability groups. However, the authors did not find significant differences between groups on depression.

Finally, Willcutt et al. (2013) found that Grade 6 students with both disabilities experienced more depression and anxiety than the single disability groups, based on parents’ diagnostic interviews. The same results were found for an internalizing problems composite score created by parents and teacher ratings. However, in Willcutt et al.’s (2013) study, the RD and RDMD groups differed in the reading achievement tests, and the MD and RDMD groups differed in the mathematics achievement tests; the RDMD group had lower scores than the RD group in reading and lower scores than the MD group in mathematics. This implies that the reported differences among groups on internalizing measures might be due to more reading/mathematics difficulties and not because of the comorbidity. In summary, the comorbidity studies assessed different internalizing problems (e.g., anxiety, depression, somatic complaints, general internalizing problems) and reported mixed results.

## The Role of Attention

Although the available literature has shown that RD, MD and internalizing problems are independently associated with attention-deficit/hyperactivity disorder (ADHD) symptoms, only a few studies have controlled for this variable and found mixed results (Arnold et al., 2005; Carroll et al., 2005; Willcutt et al., 2007; Willcutt & Pennington, 2000b, 2013). The difference between groups with and without dyslexia on depression found by Carroll et al. (2005) was not significant when inattention (measured with rating scales and structured interviews) was controlled. However, the differences between groups with and without difficulties in reading reported by Arnold et al. (2005) remained significant after controlling for this variable (measured with rating scales and structured interviews). This result is supported by other studies which found that RD is independently associated with higher rates of anxiety and depression after controlling for symptoms of ADHD and other disruptive disorders (Maughan et al., 2003; Willcutt & Pennington, 2000b). In summary, only a few studies have controlled for ADHD symptoms, and most of the studies are on reading. To our knowledge, only Willcutt et al. (2013) examined the association between different types of LD (RD, MD, RDMD, CA), ADHD and internalizing problems. Willcutt et al. (2013) subdivided their RD, MD, and RDMD groups as a function of comorbid ADHD. Their groups with RD only, MD only, and RDMD had significantly higher rates of depression regardless of whether they also had ADHD, although the group with both RD and ADHD exhibited higher rates of depression compared with the group with only RD. On the other hand, the results were mixed for anxiety: the MD with ADHD group had significantly higher rates of anxiety, while in the RD and RDMD groups, the rates of generalized anxiety disorder were similar regardless of the presence of ADHD. Even though LD is associated with the inattentive subtype of ADHD (Willcutt & Pennington, 2000a), to our knowledge, no study has controlled for attention measured with behavioral measures of attention and not rating scales. Behavioral measures, such as the number of correct responses or reaction times on cognitive tasks, provide an objective way to operationalize attention (Naglieri et al., 2014).

## The Present Study

The purpose of this study was to examine whether children with RDMD experience more internalizing problems (anxiety, depression, somatic complaints, and social withdrawal) than children with difficulties in either reading or mathematics. More specifically, we asked the following research questions:

**Research Question 1:** Do children with comorbid difficulties (RDMD) experience more internalizing problems than children with a single difficulty or children without any difficulties? In line with the multiple-deficit model, we expected that children with both RD and MD would experience significantly more anxiety, depression, somatic complaints and social withdrawal than children with either RD or MD and that children with either RD or MD would experience more internalizing problems than their chronological-age (CA) controls.
**Research Question 2:** Do group differences in internalizing problems remain significant after controlling for attention? We hypothesized that the group differences would remain significant, with children having comorbid difficulties exhibiting significantly higher levels of internalizing problems compared to those without comorbidity or any difficulties and children with either reading or mathematics difficulties.

## Method

### Participants

Sixty-one classrooms from 15 schools in Alberta, Canada, were invited to participate in this study. The teachers of these classrooms were asked to nominate students from their class who could potentially qualify for our four groups (RD, MD, RDMD, and CA) based on screening data they collected in their school or based on existing learning disabilities coding (Code 54 in Alberta) that some children already had. Two hundred and eighty students were subsequently invited to participate in further screening to form our four groups. Two hundred and forty children with parental consent were subsequently tested in two reading and two mathematics tests to confirm their group membership. Following the cut-off score recommendations used in previous research on LD (e.g., Arnold et al., 2005; Chen et al., 2023), to qualify for the RD group, the participants had to score a) below a standard score of 85 in Word Reading from the Wide Range Achievement Test-5 (WRAT-5; Wilkinson & Robertson, 2017) and Test of Word Reading Efficiency-2 (TOWRE-2; Torgesen et al., 2012) efficiency (i.e., composite score derived from Sight Word Reading Efficiency and Phonemic Decoding Efficiency; see Material section for more information), and b) above a standard score of 90 in two mathematics tests (Math Computation from WRAT-5 [Wilkinson & Robertson, 2017] and Math Fluency from the Wechsler Individual Achievement Test-3 [WIAT-3; Wechsler, 2009]; see Material section for more information). To qualify for the MD group, the participants had to score below a standard score of 85 in the mathematics tests (Math Fluency and Math Computation) and above a standard score of 90 in the reading tests (Word Reading and TOWRE). To qualify for the RDMD group, the participants had to score below a standard score of 85 in both reading and mathematics. Finally, to qualify for the CA group, the participants had to score above a standard score of 90 in both reading and mathematics. Some children with very high scores in both reading and mathematics had to be excluded for our RD or MD group to have comparable performance to the CA group in mathematics and reading, respectively (i.e., the RD group having comparable performance to the CA group in mathematics and the MD group having comparable performance to the CA group). The final sample consisted of 33 Grade 5 and 6 children with RD (51.5% female; *M*_age_ = 10.80 years, *SD* = 0.68), 35 with MD (60.0% female; *M*_age_ = 10.79 years, *SD* = 0.66), 37 with RDMD (45.9% female; *M*_age_ = 10.79 years, *SD* = 0.65), and 42 CA (64.3% female; *M*_age_ = 10.82 years, *SD* = 0.63). Forty-one of the participating children had a formal diagnosis of learning disabilities. In Canada, learning disabilities are diagnosed according to the criteria outlined in the *Diagnostic and Statistical Manual of Mental Disorders* (5th ed., text rev.; *DSM-5-TR*; APA, 2022). Sample characteristics are shown in Table 1.

**Table 1.** Sample Characteristics.

Variables	RD (*n* = 33)		MD (*n* = 35)		RDMD (*n* = 37)		CA (*n* = 42)		*F*(3,142)	*p*	η^2^	Post hoc (Bonferroni)
	*M*	*SD*	*M*	*SD*	*M*	*SD*	*M*	*SD*				
Age (year.month)	10.8	.68	10.8	.66	10.8	.65	10.8	.63	.021	.996	.000	
Gender (female percentage)	51.5%		60.0%		45.9%		64.3%					
TOWRE	81.03	4.93	99.74	8.04	77.32	4.53	102.43	6.20	165.234	.000	.776	RD = RDMD < MD = CA
WR	83.24	2.02	99.97	7.97	79.89	5.17	102.17	4.93	162.922	.000	.774	RD = RDMD < MD = CA
Math Comp.	95.88	6.07	78.74	4.89	76.76	5.03	96.50	5.30	148.833	.000	.757	MD = RDMD < RD = CA
Math Fluency	103.09	8.59	79.09	5.77	75.49	7.40	105.48	8.81	151.539	.000	.761	MD =RDMD < RD = CA
Matrices	95.03	7.31	93.51	8.77	93.41	8.28	96.19	7.99	1.046	.374	.021	RD = MD = RDMD = CA

*Note.* TOWRE = Test of Word Reading Efficiency; WR = Word Reading; Math comp. = Mathematics Computation; Math Fluency = Mathematics Fluency; RD = reading difficulties; MD = mathematics difficulties; RDMD = comorbid reading and mathematics difficulties; CA = chronological-age controls. Forty-one of the participating children had a formal diagnosis of learning disabilities.

A series of ANOVAs with group (CA, RD, MD, and RDMD) as a fixed factor showed that all groups were comparable in age, *F*(3, 142) = .021, *p* = .996, η^2^ = .000 and general cognitive ability, *F*(3, 142) = 1.046, *p* = .374, η^2^ = .021 (see Table 1). Gender distribution was also comparable across groups, χ^2^(3) = 3.179, *p* = .365. As expected, children with reading difficulties (RD, RDMD) scored lower on the reading tests compared to children without reading difficulties (MD, CA) (*p*s < .001). No significant differences were observed between the MD and CA groups, as well as between the RD and RDMD groups on the reading tests. In turn, children with mathematics difficulties (MD, RDMD) scored lower on the mathematics tests compared to children without mathematics difficulties (RD, CA) (*p*s < .001). Also, no significant differences were observed between the RD and CA groups, as well as between the MD and RDMD groups in the mathematics tests.

The teachers of the participating students also participated in the study by rating their students in internalizing problems using the Behavior Assessment System for Children, third edition (BASC-3; Reynolds & Kamphaus, 2015, see below for details).

### Materials

#### General Cognitive Ability

General cognitive ability was assessed with the Matrices task from the Cognitive Assessment System-2 (CAS-2) Brief (Naglieri et al., 2014). Children were asked to analyze the relationship between shapes and their geometric arrangements, which were interconnected through spatial or logical organization, and identify the missing component, selecting the most appropriate choice from six options. Naglieri et al. (2014) reported Cronbach’s alpha reliability to be .88.

#### Attention

Attention was assessed with the Expressive Attention task from the CAS-2-Brief (Naglieri et al., 2014). Children were first asked to read a sequence of color words (i.e., Blue, Yellow, Green, and Red) arranged in a quasi-random order in eight rows of five. Next, children were asked to name the color of a series of blocks printed as the colors in the previous page. Finally, in the last page, color words were printed in a color different from the word's name (e.g., the word green may appear in yellow ink), and children were asked to name the color of the ink in which the word was printed (e.g., Blue appearing in red ink is read as “Red”). The time and accuracy of the last task were recorded and combined to obtain a ratio score. The ratio score was then converted to a scaled score. Naglieri et al. (2014) reported Cronbach’s alpha reliability to be .89.

#### Reading

Reading was assessed with two tests: the Word Reading task from the WRAT-5 (Wilkinson & Robertson, 2017) and the TOWRE-2 task (Torgesen et al., 2012) that includes two sub-tasks (the Sight Word Reading Efficiency and the Phonemic Decoding Efficiency). In Word Reading, children were asked to read words arranged in terms of increasing difficulty. The task was discontinued after five consecutive errors and the maximum score was 70. Wilkinson and Robertson (2017) reported split-half reliability for ages 10 and 11 to be .93 and .95, respectively. In Sight Word Reading Efficiency, children were asked to read as fast as possible a list of real words of increasing difficulty (max = 108). In Phonemic Decoding Efficiency, children were asked to read as fast as possible a list of nonwords (max = 66). In both tasks, a participant's score was the total number correct within 45 seconds. Following the instructions in the manual, the raw scores from Sight Word Reading Efficiency and Phonemic Decoding Efficiency were converted to a scaled score and then summed together to obtain a composite (standard) score that was used to determine if the children qualified or not for our groupings. Torgesen et al. (2012) reported alternate forms reliability to be .91 for Sight Word Reading Efficiency and .92 for Phonemic Decoding Efficiency.

#### Mathematics

Mathematics was assessed with two tests: Math Computation from the WRAT-5 (Wilkinson & Robertson, 2017) and Math Fluency from the WIAT-3 (Wechsler, 2009). In Math Computation, children were asked to solve as many calculations as possible within a 15-minute time limit, and the maximum score was 55. Wilkinson and Robertson (2017) reported split-half reliability for ages 10 and 11 to be .95 and .91, respectively. In Math Fluency, children were asked to solve as many written addition (max = 48), subtraction (max = 48), and multiplication problems (max = 40) as possible within a 60-second time limit for each type of problem. In the three tasks, a participant's score was the total number correct. Following the instructions in the manual, the raw scores from addition, subtraction and multiplication were converted to a scaled score and then summed together to obtain a composite score for mathematics fluency. Wechsler (2009) reported test-retest reliability to be .84 for addition, .89 for subtraction, and .90 for multiplication.

#### Internalizing Problems

Internalizing problems were assessed using the anxiety, depression, somatic complaints and withdrawal teacher-rating scales (ages 6–11) from the BASC-3 (Reynolds & Kamphaus, 2015). These are four-point rating scales (never to almost always) with statements about students' anxiety (e.g., “appears tense,” “is easily stressed,” “worries about things that cannot be changed”), depression (e.g., “cries easily,” “is sad,” “is negative about things”), somatic complaints (e.g., “complains about health,” “complains of pain,” “is afraid of getting sick”) and withdrawal (e.g., “has trouble making new friends,” “avoids other children,” “avoids making friends”) behaviors. There were nine statements for anxiety, 11 statements for depression, eight statements for somatic complaints and eight statements for withdrawal. Reynolds and Kamphaus (2015) reported Cronbach’s alpha reliability for anxiety, depression, somatic complaints and withdrawal scales to be .86, .86, .87, and .90, respectively.

### Procedure

The academic achievement, attention and general cognitive ability tests were administered individually by trained research assistants in a quiet room in two 20-minute sessions. Ethics approval was granted by the Research Ethics Board of the University of Alberta (Pro00130649). Informed consent was obtained from parents, and approval was also received from the school principals and teachers. The teachers also signed a consent form prior to completing the internalizing problems scale. Finally, students provided their oral assent prior to testing, ensuring they understood the nature of the activities and their right to withdraw at any time.

## Results

Before conducting any group comparisons in internalizing problems, we examined if the assumptions of the analysis of variance (ANOVA) were met. The assumption of normality was violated in all variables, and the assumption of homogeneity of variance was violated in the depression and somatic complaints variables. Although ANOVAs are robust to normality deviations (Blanca et al., 2017), we also performed Kruskal–Wallis tests (Hecke, 2012) to confirm the results. Four ANOVAs with and without attention as a covariate were conducted to examine group differences in internalizing problems. Post hoc testing was corrected using Bonferroni correction. Effect sizes (η^2^) were computed for all tasks. Cohen (1988) provided benchmarks for effect sizes, defining small effects (η^2^ = .01), medium (η^2^ = .06), and large (η^2^ = .14) effects. The results from all ANOVAs are presented in Table 2.

**Table 2.** Group Comparisons on Each Internalizing Problem.

Problem	RD (*n* = 33)		MD (*n* = 35)		RDMD (*n* = 37)		CA (*n* = 42)		ANOVAs			
	*M*	*SD*	*M*	*SD*	*M*	*SD*	*M*	*SD*	*F*(3, 143)	MSE	η^2^	Post hoc
Anxiety	51.33	9.88	54.60	12.79	57.86	12.80	50.57	11.12	3.061*	137.4	.06	RDMD > CA RD = MD = RDMD RD = MD = CA
Depression	49.97	11.13	54.51	15.34	55.19	12.05	47.52	9.20	3.595*	144.5	.07	RDMD > CA RD = MD = RDMD RD = MD = CA
Somatic complaints	48.18	8.08	48.86	9.39	49.05	6.86	45.81	5.08	1.625	54.9	.03	RD = MD=RDMD = CA
Social withdrawal	51.82	9.07	51.69	8.71	52.51	10.43	48.79	7.41	1.373	79.6	.03	RD = MD = RDMD = CA

*Note*. RD = reading difficulties; MD = mathematics difficulties; RDMD = comorbid reading and mathematics difficulties; CA = chronological-age controls; MSE = mean squared error.

* *p* < .05.

The results of ANOVA for anxiety showed a significant main effect of group, *F*(3, 143) = 3.061, *p* = .030, η^2^ = .06. The results of the Kruskal–Wallis test also revealed a significant effect of group (Kruskal–Wallis *H* = 8.214, *p* = .042). Post hoc comparisons revealed that the RDMD group obtained a significantly higher score than the CA group (*p* = .039). There were no significant differences between the RDMD and MD groups as well as between the RDMD and RD groups. The results of ANOVA for depression also yielded a significant main effect of group, *F*(3, 143) = 3.595, *p* = .015, η^2^ = .07. The Kruskal–Wallis test was also significant (Kruskal–Wallis *H* = 11.342, *p* = .010). Post hoc comparisons showed that the RDMD group obtained a significantly higher score than the CA group (*p* = .032). There were no significant differences between the RDMD and MD groups as well as between the RDMD and RD groups. The results of ANOVA for somatic complaints and withdrawal did not reach significance (somatic complaints: *F*(3, 143) = 1.625, *p* = .186, η^2^ = .03; withdrawal: *F*(3, 143) = 1.373, *p* = .253, η^2^ = .03). The result of the Kruskal–Wallis test for somatic complaints and withdrawal did not show a significant effect of group either (somatic complaints: Kruskal–Wallis *H* = 6.393, *p* = .094; withdrawal: Kruskal–Wallis *H* = 4.089, *p* = .252). The results of ANCOVAs (using attention as a covariate) showed that the group differences did not remain significant for both anxiety (*F*(3, 142) = 2.061, *p* = .108, η^2^_p_ = .04) and depression (*F*(3, 142) = 1.974, *p* = .121, η^2^_p_ = .04). To summarize, compared to the CA group, the RDMD group exhibited higher scores in anxiety and depression. However, after controlling for attention, the group differences in anxiety and depression were no longer significant.

## Discussion

This study aimed to examine whether children with comorbid difficulties (RDMD) experience more internalizing problems than children without any difficulties or with a single difficulty. Our hypothesis that children with RDMD would experience significantly more anxiety, depression, somatic complaints and social withdrawal than the other groups was partially supported. More specifically, our results revealed group differences in anxiety and depression with a medium effect size for both anxiety (η^2^ = .06) and depression (η^2^ = .07) (Cohen, 1988). These effect sizes fall within the moderate range commonly found in the literature on the association of LD with internalizing problems (e.g., *d* = 0.61 in Nelson & Harwood, 2011, meta-analysis; *g* = -.54 in Vieira et al., 2024, meta-analysis).

Further analysis showed that only the RDMD group significantly differed from individuals without LD, although the single-domain groups also exhibited higher scores in all internalizing problems measures compared to children without difficulties, similar to what Martínez and Semrud-Clikeman (2004) found for depression. In contrast to Willcutt et al.’s (2013) study, our findings indicated that children with comorbidity in reading and mathematics did not differ from children having either reading or mathematics difficulties, although the comorbidity group exhibited higher scores. This suggests that the comorbidity RDMD is not a third disorder that is distinct from RD or MD, and their additive difficulties may contribute to internalizing problems, corroborating with the multiple-deficit framework (e.g., McGrath et al., 2020; Pennington, 2006).

These findings provide evidence that children with LD, especially those with more than one difficulty type, may be qualitatively different from children without any difficulty in terms of how they experience internalizing problems, which may have significant implications for early intervention (Neil & Christensen, 2009). Regarding the association between LD and anxiety, studies have shown that it is bidirectional (e.g., Carey et al., 2016; McArthur, 2022). According to McArthur (2022), reading difficulties can trigger anxiety, with the latter then exacerbating the former, creating a vicious cycle once this relation is established. Similarly, Carey et al. (2016) suggested that the relation between mathematics performance and mathematics anxiety is bidirectional. However, further research is needed to explore the relation between mathematics difficulties and other types of anxiety to determine if this bidirectional relation applies to them as well. On the other hand, research indicates that the association of LD with depression might be more complex than the LD-anxiety relation (Francis et al., 2019; Maughan & Carroll, 2006) and might depend on other variables such as inattentiveness (Carroll et al., 2005).

Our difference between the RDMD and CA groups was not significant after controlling for attention, corroborating previous findings that ADHD accounts for associations between LD and other disorders (e.g., Aro et al., 2024; Carroll et al., 2005; Willcutt et al., 2007). However, our findings contrast with those of Willcutt et al. (2013), where their RDMD group showed significantly elevated levels of depression and anxiety regardless of comorbid ADHD status. The fact that Willcutt et al. (2013) used a scale to measure ADHD and we tested attention with a behavioral task might have contributed to this difference. Rating scales might be easier to collect data but they are also more prone to informant biases. Therefore, our findings suggest that attention might play a role in the association of LD with internalizing problems, aligning with previous research showing that this relation is associated with the inattentive subtype of ADHD (e.g., Aro et al., 2024; Willcutt & Pennington, 2000a). Our results also corroborate the multiple-deficit neuropsychological model (McGrath et al., 2020; Pennington, 2006) that indicates that RDMD shares general risk factors, such as attention. Our results suggest that this shared risk factor may contribute to elevated anxiety and depression problems in children with LD (Georgiou & Parrila, 2023; Maughan & Carroll, 2006).

Differences between groups on somatic complaints and social withdrawal were not significant, which is different from what we expected. We expected that group differences in social withdrawal would also be significant due to previous research that found that individuals with LD presented higher scores on social withdrawal when compared to anxiety and somatic complaints (Vieira et al., 2024). One possible explanation for these non-significant differences could be that teachers may not have as many opportunities to observe the specific behaviors associated with these constructs. Although this is not a consensus, some studies indicate that parents tend to report more internalizing problems in children compared to teachers (e.g., Dahle et al., 2011; Klein et al., 2019). Parents might be more likely to notice if their child is socially withdrawn during family gatherings or playdates at home, and somatic complaints such as headaches might be more apparent during morning routines or after-school activities, whereas teachers might not be aware unless the child explicitly mentions it. However, although social withdrawal and somatic complaints were not significant, the comorbidity group had the highest scores compared to children without LD. This suggests that both constructs might be related to learning difficulties. Given that social withdrawal may be a behavioral manifestation of social anxiety (Rubin et al., 2009) and somatic complaints can be seen as a manifestation of academic work stress (Willcutt & Pennington, 2000b), these connections might be related to anxiety. This calls for more research exploring types of anxiety, such as social and academic anxiety.

### Implications for Research and Practice

Our findings have several important implications. In regard to research, our findings suggest that RDMD should not be treated as a separate condition but rather as the compounded effect of RD and MD. This aligns with the propositions of the multiple-deficit framework, according to which learning difficulties arise from the interaction of domain-specific and shared risk factors, resulting in an additive profile of combined deficits (McGrath et al., 2020; Pennington, 2006). Second, they suggest that we need to be specific when we refer to the internalizing problems of children with RD, MD or RDMD as children in these groups may not differ from their CA controls in all types of internalizing problems.

In terms of practice, the fact that our group differences were not significant after controlling for attention highlights the importance of incorporating attention measures into the evaluation process for internalizing problems in children with LD. Developing integrated assessment tools would enhance both research and clinical practice, offering a more comprehensive understanding of students’ needs. Our results also suggest that interventions that strengthen executive attention or self-regulation could help in addressing both LD and internalizing problems, such as the Tools of The Mind Program for preschoolers (Diamond et al., 2007). In this program, students develop the skills to set meaningful learning goals and evaluate their achievement, which involves focused attention, purposeful reflection, effective memory use, and the ability to shift focus between different aspects of a learning task.

### Limitations

Some limitations of the present study are worth noting. First, even though there is evidence that externalizing problems are related to LD (e.g., Kempe et al., 2011; Willcutt & Pennington, 2000b), we did not examine externalizing problems in this study. Instead, we focused on exploring the connections between internalizing problems and LD that have been historically understudied. Given the already documented evidence for positive associations between youth externalizing problems and gaps in reading and mathematics, we excluded externalizing problems from further study for practical reasons. Our decision to exclude externalizing problems was also based on our previous experience working with teachers telling us that, because of their heavy workload, they are less willing to fill out long surveys; our questionnaire already had 36 items. Second, only 41 of our participants had a formal diagnosis of learning disabilities and we did not find any information from the school if any of them had received any intervention for their learning difficulties or internalizing problems. Third, we used only teacher ratings of children’s internalizing problems. Our choice was deliberate because, in the past, we did not have a good return rate for parent questionnaires, and we were concerned that adding self-reports would extend children’s assessments, potentially leading to exhaustion and stress. Fourth, our study included only Grade 5 and 6 children and our findings may not generalize to other grade levels. Fifth, although we employed a frequently used measure of attention, it only measures verbal inhibition, which is one component of attention related to its executive function (Eysenck & Derakshan, 2011). Future studies may consider adding measures of alerting and orienting response, following the attention network theory (Petersen & Posner, 2012). Finally, although the study controlled for attention as a potential confounding variable, there may be other variables (e.g., self-concept) that may also influence the relation between comorbid learning difficulties and internalizing problems that we did not include in our study.

### Conclusion

To conclude, our findings add to a small body of previous studies on the relation between comorbidity in RDMD and internalizing problems (Aro et al., 2022; Martínez & Semrud-Clikeman, 2004; White et al., 1992; Willcutt et al., 2013) by showing that children with RDMD experience higher levels of anxiety and depression compared to children without LD. Even though significant differences in somatic complaints and social withdrawal among groups were absent, the comorbidity group exhibited the highest scores on all internalizing measures. These findings underscore the complexity of internalizing problems in children with LD, suggesting that the additive difficulties in RDMD may contribute to elevated anxiety and depression. The group differences in anxiety and depression were no longer significant when attention was controlled, indicating that this shared risk factor plays a role in this association. An obvious implication of this finding is that if we address possible attention problems in children with LD, their anxiety and depression may also decrease.

## Notes

1. In this study, we use the term “learning difficulties” to acknowledge the broader range of academic struggles, as not all participants in this study received a formal learning disability diagnosis; throughout the article LD = learning difficulties.
